# Hospital Meetings

**Published:** 1903-04-18

**Authors:** 


### HOSPITAL MEETINGS.

### HAMPSTEAD GENERAL HOSPITAL.

Mr. W. F. Malcolm took the chair at the annual meeting of this hospital held on April 4th, when the attendance was only of a moderate description. Commenting on the report for the past year he said that it showed what good work had been done by the hospital in spite of the inadequacy of the accommodation. The financial position was most promising, but it should be borne in mind that during last year the donations and legacies were much larger than ever before, and as these were fluctuating items they would have to exercise great care to maintain the hospital in its present prosperous condition. The annual subscriptions, which were the mainstay of the hospital, had advanced and it was much hoped that at the end of the present year their total would be well into four figures. They had received grants from the Sunday, Saturday, and King Edward's Funds. The collections in churches had however fallen off. The chairman then referred to the resignation of the hon. secretary, Mr. R. A. Owthwaite, who had been connected with the hospital for 21 years, since its first establishment, and paid a tribute to his excellent work. In October 1902, H.R.H. Princess Christian laid the foundation stone of the new building which it was earnestly hoped would be ready for occupation by 1904. Mr. Malcolm ended by an appeal to the inhabitants of Hampstead to furnish them with the funds requisite for the carrying on of the great work of building their new hospital.

A medical report was read to the meeting, after which an eloquent tribute to the splendid work done by the medical staff was paid by Mr. F. Macmillan.

The report of the Ladies' Association having been read and the customary business proceeded with, the meeting became an extraordinary general one in order to consider two resolutions, to the effect that the name should be changed from the Hampstead Home Hospital and Nursing Institute to the Hampstead General Hospital; and that necessary alterations and amendments should be made in the articles of association.

The necessity for the change of name was briefly explained by Mr. E. K. Blyth, who said that the hospital had developed largely since its foundation twenty-one years ago, when it was mainly devoted to private nursing and to providing nurses. After some discussion the resolutions were passed unanimously, and the proceedings terminated.

